# A shedding analysis after AAV8 CNS injection revealed fragmented viral DNA without evidence of functional AAV particles in mice

**DOI:** 10.1038/s41434-024-00447-z

**Published:** 2024-03-12

**Authors:** Felix Krause, Katja Schmidtke, Mailton Franca de Vasconcelos, David Schmidt, Beyza Cansiz, Franziska Theisen, Melanie D. Mark, Max O. Rybarski

**Affiliations:** Department of Behavioral Neuroscience, ND7/31, Ruhr-University Bochum, Universitaetsstr. 150, D-44780 Bochum, Germany

**Keywords:** Gene expression analysis, Gene therapy

## Abstract

Adeno-associated viruses (AAV) are commonly used in the scientific field due to their diverse application range. However, AAV shedding, the release of virions from the host organism, can impact the safety of AAV-based approaches. An increasing number of authorities require the characterization of vector shedding in clinical trials. Recently, shedding of transduced laboratory animals has also gained attention regarding the necessary disposal measures of their waste products. However, no explicit international regulations for AAV-shedding waste exist. Generating insights into shedding dynamics becomes increasingly relevant to help authorities develop adequate regulations. To date, knowledge of AAV vector shedding in mice is very limited. Moreover, confirmation of functional shed AAV particles in mice is missing. Therefore, we examined feces, urine, and saliva of mice after CNS injection with AAV2/8. It revealed the presence of viral DNA fragments via qPCR for up to 4 days after injection. To examine AAV functionality we performed nested PCR and could not detect full-length viral genomes in any but two collected feces samples. Furthermore, a functional infection assay did not reveal evidence of intact AAV particles. Our findings are supposed to contribute murine shedding data as a foundation to help establish still lacking adequate biosafety regulations in the context of AAV shedding.

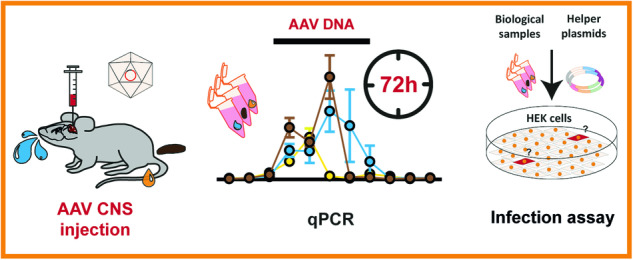

## Introduction

Adeno-associated viruses (AAVs) are an important tool in human therapy and research. They facilitate gene delivery to cells with low immunogenicity and long-term transgene expression [1, 2], being generally well tolerated [3]. Since their use in clinical trials, there has been a growing concern that AAV particles may be shed into the environment with unknown consequences [4]. Recently, AAV shedding in animal research has gained attention from different authorities [5]. There is evidence that transduced organisms can shed functional particles to some extent depending on the application route and viral load [4, 6]. The resulting waste products (i.e., bedding material) could be considered potentially hazardous requiring adequate inactivation before disposal. To date, there are no specific, unified international rules for the handling of this material. The European Union does not specifically mention the waste products of transduced animals but classifies recombinant AAVs as genetically modified organisms (GMO), requiring inactivation before disposal. Consequently, the treatment of these waste products depends on the presence of functional AAV particles. However, the cautionary principle applies to any potentially GMO-contaminated material in absence of evidence, often requiring the bedding material of transduced animals to be autoclaved, as stated in the Council directive 2001/18/EC(2001) [7]. In accordance with this principle, the German authorities require waste products of AAV-transduced animals to be treated as GMO-contaminated for a period they assume to be adequate (usually 7 days after AAV transduction) unless data are provided indicating otherwise [8]. Lacking information regarding the duration and biological significance of shedding results in the absence of uniform, and sometimes too harsh regulations. Since adequate waste disposal is time consuming and expensive, the necessity of shedding analyses in laboratories has increased to reduce research costs and/or fulfill regional regulations. The growing concern of AAV risk and regulations paired with the widespread use of AAVs in the research community, makes the characterization of the AAV shedding profiles increasingly relevant.

Although CNS injection of AAVs is regularly performed in neuroscientific animal laboratories, the knowledge about resulting AAV shedding is very limited [9, 10], while data from mice are lacking completely. Therefore, we investigated the AAV2/8 shedding dynamics after stereotactic CNS injections (cerebellum) in mice. Biological samples (urine and feces) were collected for up to 6 weeks as primary shedding sources due to their waste-removal function and high output mass/volume. Saliva was included due to its risk to transfer virions directly to the human body through bites from transduced mice. Quantitative real-time PCR, a common method to examine AAV shedding [6] was applied to identify biofluids/excreta containing AAV-DNA. While qPCR is a sensitive technique, it is not able to differentiate between infectious and non-infectious viral particles potentially causing false-positive results and not allowing an accurate risk assessment. Conclusively, it is desirable to assess the infectivity of shed AAV DNA with a functional assay. The most used method requires a pathogenic Adenovirus and therefore an active BSL-2 laboratory. However, the increasing regulatory demand for AAV shedding analyses could pose challenges to workgroups without access to a BSL-2 laboratory. One of the main advantages of AAVs in a non-clinical context is their BSL-1 classification and the potential necessity to perform BSL-2 experiments to work with AAVs would greatly diminish their use case. Therefore, we designed a BSL-1 compatible infection assay to provide an easily accessible tool for animal laboratories to perform functional shedding analysis to deal with regulatory demands until unified, international regulations are in place.

We aimed to address the functionality of shed AAV2/8 particles in qPCR-positive samples collected after intracerebellar AAV injections. First, we determined the amount of full-length AAV genomes shed using nested PCR. Secondly, we applied a BSL-1 compatible infection assay utilizing helper plasmids instead of an adenovirus to aid in the amplification of full-length intact AAV particles to optimize the amplification of minuscule levels of AAV particles before detection.

## Materials and methods

### Mice

Six female 12-month-old C57/Bl6J mice (JAX stock #:000664) were single housed on a 12 h light/dark at 22–24 °C with *ad libitum* access to food and water. The present study was carried out in accordance with the European Communities Council Directive of 2010 (2010/63/EU) for care of laboratory animals and approved by a local ethics committee (Bezirksamt Arnsberg) and the animal care committee of North Rhine-Westphalia, Germany. The study was supervised by the Animal Welfare Commission of the Ruhr-University Bochum.

### AAV vector production

The AAV2/8-CMV-mCherry was produced using HEK293T cells (Sigma Aldrich 12022001; no recent mycoplasma screening) utilizing the three-plasmid system with polyethylenimine as previously described [11]. We performed a qPCR dilution series with pAAV-CMV-mCherry DNA (10^0^–10^9^ calculated copies) for titer determination (Fig. S1A). The quantitation limit of the reaction was determined at ten DNA copies. The precision was adequate (*R*^2^ = 0.9936), resulting in the standard curve equation: *y* = −3.137*x* + 37.452. A virus titer of 2.1 × 10^9^ genomes/µl was calculated.

### Intracranial injections

A sagittal incision along the cranial midline was performed. The craniotomy was performed 5.9 mm from bregma and 2 mm towards the left hemisphere. The AAV2/8-CMV-mCherry (8.4 × 10^8^ copies) was pressure injected into the cerebellum from 1.8 to 1.4 mm depth in 100 µm steps with 2 min incubations between injections [11].

### Sample collection

The sample collection was performed as described by the workgroup of Le Guiner under sterile conditions [12]. Mice were individually placed in sterilized cages without bedding and one fresh feces pellet, a saliva swab, and 120 µl of urine were collected per mouse and resuspended in growth medium followed by vortexing, centrifugation (900 × *g*, 5 min, RT), filtering (0.22 µm) and storage at −80 °C. The DNA extraction was performed using the QIAamp Viral RNA Mini Kit (Qiagen, Hilden, Germany) according to manufacturer’s protocol. To increase DNA yield the elution step was repeated resulting in 80 µl eluate. One sample was excluded from the analysis due to contamination during the sample collection (saliva: M2, 72 h).

### Infection assay

To test the infectivity of shed AAV DNA from collected biological samples, we designed a helper virus-free, BSL-1 compatible version of commonly used infection assays, based on the three-plasmid system [13–16] allowing AAVs to replicate in HEK293T cells in presence of helper plasmids to increase their abundance prior to qPCR detection. In brief, 7.5 × 10^4^ low passage HEK293T cells were seeded in 24-well plates. After 18 h of incubation, cells were transfected with pRep2/Cap8-plasmids (68 ng/well) and p-helper-plasmids (82 ng/well) using PEI. The collected samples were added 6 h after transfection and incubated for 72 h. The DNA of the supernatant (140 µl) was extracted with the QIAamp Viral RNA Mini Kit and from cells with the DNeasy Blood and Tissue Kit (Qiagen, Hilden, Germany) and quantified with a NanoDrop^TM^2000. The sensitivity of the assay was determined using a dilution series of functional AAV particles ranging from 2 × 10^5^ to 0 as input/well followed by qPCR quantification.

### Quantitative real-time PCR

The used primer and probe sequences were validated against the vector expression cassette: 5′-forward-primer: GTCCAAGCTAGGCCCTTTTG, 3′-reverse-primer: GCTTCAAGGTGCACATGGA and Taqman-Probe: 5′-FAM-CGAGGAGGATAACATGGCCA-TAMRA-3′. All reactions were set up with a volume of 20 µl: 1xGo Taq Probe qPCR Mastermix (Promega, Madison, Wisconsin, USA) 0.9 µM per primer, 2 µM probe, 5 µL template (supernatant samples) or 100 ng DNA (cellular samples). The qPCR reactions consisted of denaturation at 95 °C for 2 min and 40 cycles of denaturing for 15 s at 95 °C combined with annealing and extension for 60 s at 60 °C. The cycle threshold values were generated with the Rotor GeneQ series Software 2.3.5 (Qiagen, Hilden, Germany). A standard curve was generated from a dilution series (10^8^–0.1 copies) and an efficiency between 0.95–1.1 and vector copies/reaction using the formula: 10^((CT−37.452)/−3.137) were calculated. Every qPCR sample set included non-template controls and samples with known AAV copy numbers.

Biodistribution analysis of the liver was performed with the BRYT green® Dye (Promega, Madison, Wisconsin, USA) and normalized with amplicons of genomic DNA (Chr. 10, product size: 270 bp) with the primers 5′-TTGTTATGTGGGTCCTGCGG-3′ and 3′-GTAGAAGCCCTCAGTCCTCG-5′. Specificity of obtained signals was determined with gel electrophoresis. One sample displaying unspecific qPCR signal was removed from the analysis (saliva 72 h).

### Nested polymerase chain reaction

A nested polymerase chain reaction (PCR) was performed to detect minuscule amounts of AAV DNA using the GoTaq Hot Start Mastermix Green (Promega, Madison, Wisconsin, USA). 0.5 µM per primer and 100 ng of DNA from the biological samples were applied. The first PCR used the primer 5′-TCACTAGGGGTTCCTGCGG-3′ located in the inverted terminal repeat region of the AAV genome. The Robocycler96s program consisted of initial denaturation at 94 °C for 3 min and 40 cycles of denaturing for 30 s at 94 °C, annealing for 30 s at 55 °C and extension at 72 °C for 3 min. A final extension step was performed at 72 °C for 3 min. Subsequently, the samples were diluted 1:20 and subjected to additional 35 cycles of PCR with the same parameters using 0.5 µM of the primers 5′-ATTACGGGGTCATTAGTTCA-3′ and 3′-GCACGTGGTTACCTACAAA-5′ located at the edges of the mCherry insert before visualization by gel-electrophoresis. Two samples (feces 24 h, saliva 24 h) were used up before the final analysis and are therefore not included in the reported data set reducing the sample size for those data points from six to five.

### Statistical analysis

The statistical analyses were performed with SigmaPlot (14.0) and GPower (3.1.9.4). A sample size of six was calculated a priori assuming a moderate effect size of 0.4 leading to a power prediction of 0.96. The error bars display mean ± SEM. Statistical significance is reported as follows: *p < 0.05; **p < 0.01; ***p ≤ 0.001. The generated data were tested for normal distribution and equal variance to apply the appropriate statistical tests. Animals were not randomized since only one experimental group was present, but the samples for the subsequent molecular analysis were randomized by encoded labeling.

## Results

### AAV-DNA was detected in feces, urine, and saliva of mice primarily between 24–72 h after intracranial injection

**Fig. 1:**
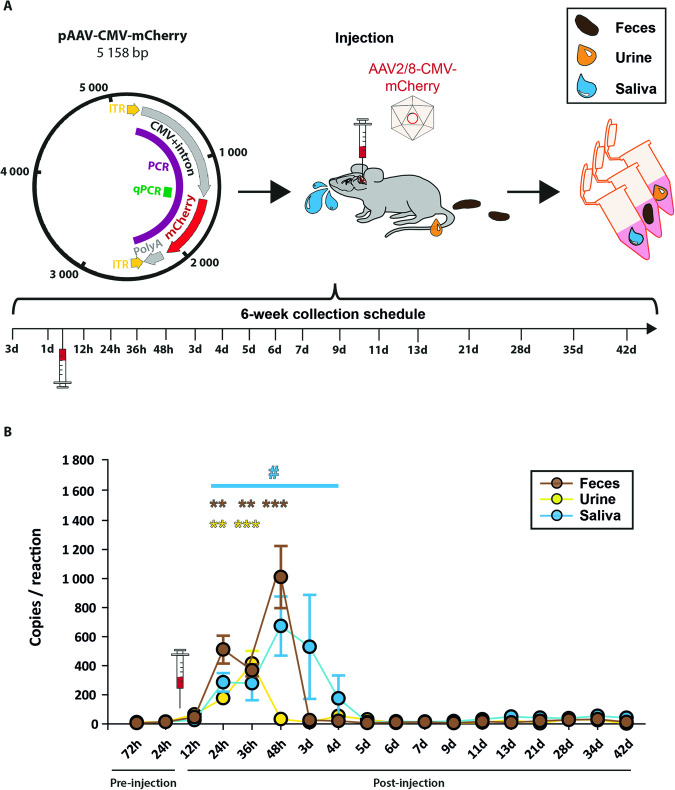
Detection of viral DNA in feces, urine, and saliva up to 72 h after CNS AAV injection in mice. **A** Plasmid map of the utilized pAAV-CMV-mCherry with target regions for qPCR (201 bp, green) and nested PCR (2612 bp/2448 bp, magenta). 8.4 × 10^8^ vector copies (vc) were injected in the cerebellar region of six mice. Feces (brown), urine (yellow), and saliva (blue) samples were collected as displayed in the schedule. **B** qPCR of collected samples detected vc above baseline values 12–96 h post injection (127–1406 vc/reaction). Data are reported as mean ± SEM. Statistical significance was evaluated by one-way RM (repeated measure) ANOVA, post hoc test: Dunnet’s method (*n* = 6): urine *p* = 0.005 (24 h), *p* = 0.001 (36 h); feces *p* = 0.009 (24 h), *p* = 0.027 (36 h), *p* = 0.001 (48 h); saliva *p* = 0.072.

To examine AAV shedding in mice after CNS injection, virus functionality was confirmed by injection of 8.4 × 10^8^ AAV copies in mouse cerebellum, resulting in a wide-spread mCherry expression in Purkinje cells after 7 days (Fig. S1B). Control samples were gathered 72 h and 24 h before AAV injection to establish baseline values. Biological samples were obtained for 6 weeks with decreasing frequency, focusing mainly on the first 48 h after injection (Fig. 1A). AAV genomes in cerebellar tissue of all animals were detected via qPCR. Although one mouse (M1) displayed a reduced copy number (Fig. S1D), its shedding profile was not visibly different compared to the other animals, indicating a deviation of the injection spot instead of unsuccessful virus application. Therefore, the mouse was included in the data set. qPCR can only amplify small AAV-DNA fragments (202 bp), thereby potentially including AAV-DNA debris. To verify the expression of full-length AAV genomes (product: 2612 bp) from injected cerebella, a PCR was performed using a primer targeting the inverted terminal repeat region of the AAV genome (Fig. S1C). Specificity was confirmed by sequencing (data not shown). AAV genomes were detected in all animals, while M1 also displayed a weaker expression (Fig. S1D). However, the expression of full-length- to shorter qPCR AAV-products was not comparative indicating the presence of AAV-DNA fragments. To control for qPCR-inhibition a fixed amount of functional AAV-DNA copies (1.5 × 10^7^) in the presence or absence of 100 ng isolated DNA from pre-injection biological samples were compared with qPCR (Fig. S1E). No impairments in qPCR were measured (ANOVA on ranks, *p* = 0.664).

After establishing qPCR/PCR conditions, samples were monitored for the presence of AAV-DNA (Fig. 1B). Preinjection samples only showed minuscule values near the sensitivity limit of the qPCR reaction (feces: 7.7 ± 6.15; urine: 12 ± 8,9; saliva: 10 ± 6.26) (Fig. 1B). In contrast, urine samples 24 h after injection displayed increases of AAV-DNA, peaking after 36 h with up to 762 detected copies/reaction (415 ± 79) equating to 791 ± 151 copies of AAV DNA per microliter urine. All later time points did not differ from the baseline. Similar increases in AAV-DNA were observed from fecal samples, exhibiting peak shedding after 48 h with up to 1528 copies/reaction (1010 ± 195) followed by a return to baseline. This translates to an average of 9684 ± 1869 copies of AAV DNA per milligram of fecal matter. A prolonged shedding of DNA was detected in saliva samples between 24 h and 4 days after injection, reaching its maximum after 48 h with up to 1872 copies/reaction (673 ± 186) indicating an average of 153,913 ± 42,427 AAV DNA copies per swab. Together, these results confirm the presence of shed AAV DNA in the collected samples, mainly 48–72 h after CNS injection.

### Full-length AAV genomes could only be detected in a small subset of collected feces samples

**Fig. 2:**
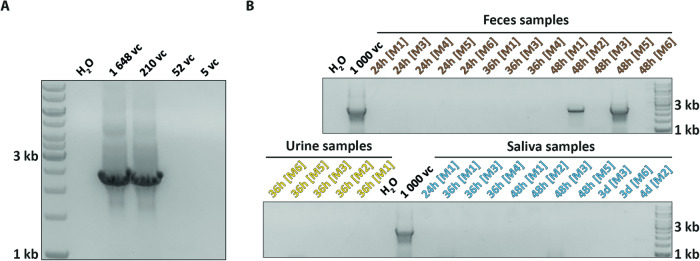
Detection of full-length AAV-DNA in two qPCR-positive feces samples 48 h post AAV injection. **A** Gel image of full-length AAV PCR products (2448 bp) from a dilution series of DNA isolated from an AAV vector stock that was quantified by a qPCR standard curve. The PCR reliably produced a band at the expected weight, and its specificity was confirmed by sequencing (data not shown). Bands were reliably visible down to 210 AAV vector genomes as an input. **B** Collected feces (*n* = 13), urine (*n* = 5), and saliva (*n* = 11) samples from six injected mice (M1-6) which displayed an elevated AAV-sequence copy numbers ranging from 342 to 1872 copies by qPCR were tested for full-length AAV DNA with a nested PCR. Positive control for specificity and sensitivity: 1000 AAV vector copies (vc). Full-length AAV genomes could not be detected in any urine or saliva sample in spite of inputs above the sensitivity limit (bottom), indicating a high degree of DNA fragmentation of shed AAV likely containing less than 210 full-length genomes. The nested PCR produced specific bands in two feces samples collected 48 h after AAV injection (top) indicating the presence of full-length AAV genomes exceeding 210 vector copies.

To investigate whether samples containing AAV-DNA sequences identified by qPCR represent intact AAV genomes, they were tested by nested PCRs with primers amplifying almost the entire AAV genome (Fig. 1A). A dilution series of AAV-DNA isolated from our vector stock was conducted revealing a PCR sensitivity below 1000 vector copies (Fig. S1A). Subsequently, different AAV DNA dilutions near the sensitivity limit were quantified with qPCR revealing that the necessary AAV genome input to produce a visible product is less than 210 AAV input copies (Fig. 2A). We tested samples containing more than 300 AAV-DNA copies/qPCR reaction applying the same amount in the PCR (341–1872 vc) thereby superceeding the established detection limit. Despite detecting the positive control (1000 vc), no specific full-length AAV product was generated in almost all of our tested samples indicating a high fragmentation of contained AAV-DNA. However, in two feces samples collected 48 h after AAV injection a PCR product of the correct size could be detected indicating the presence of full-length AAV genomes, most likely superceeding 52 copies per reaction.

### A functional, BSL-1 compatible infection assay did not detect AAV replication in collected samples

**Fig. 3:**
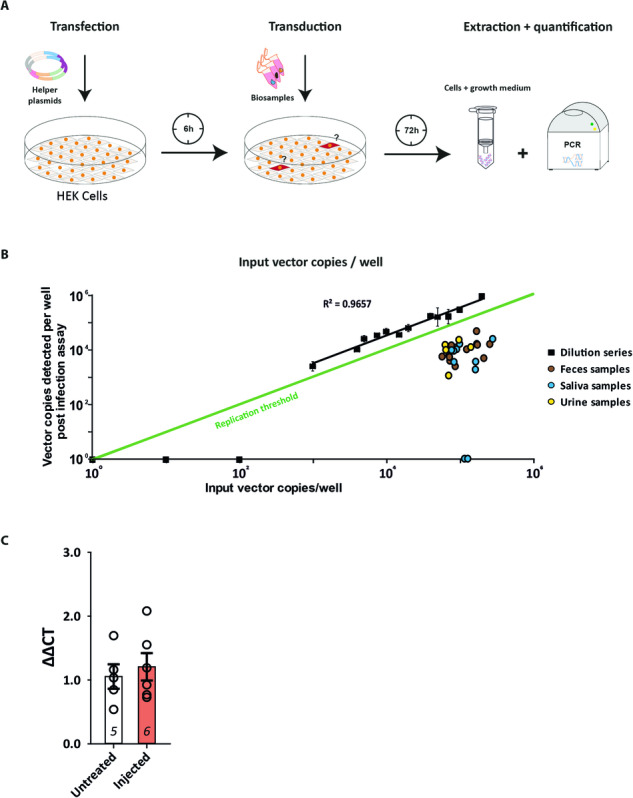
The functional infection assay did not reveal viral replication in the tested biological samples. **A** Schematic of the functional infection assay. 7.5 × 10^4^ HEK293T cells were seeded 24 h before transfection. P-Helper and RC8 plasmids were applied to support AAV replication. After 6 h, filtered biological samples were added to the cells followed by 72 h replication. Cells and growth medium were harvested, and the quantity of viral genomes were compared to the input copies by PCR. **B** The sensitivity limit of the assay lies below 4 × 10^3^ input vc (Fig. S3F, G). The linear range was between 1 × 10^3^ and 2 × 10^5^ AAV input copies (black squares, *R*^2^ = 0.9657). qPCR-positive biological samples (0–46,597 vc) were subjected to the infection assay (feces, brown circles; saliva, blue circles; urine, yellow circles). Detected vc/well: feces: 11,892 ± 3091, *n* = 13; urine: 10,070 ± 3126, *n* = 5; saliva 7787 ± 2330, *n* = 10. No quantified sample revealed a proof of replication (above green line). Instead, a decrease was observed (feces: 9.66% ± 1.87%; urine: 14.54% ± 3.82%; saliva 6.76% ± 1.78%), in agreement with our control experiment (Fig. S3E). **C** AAV biodistribution from the liver of untreated (white bar) and AAV-mCherry injected (orange bar) mice after 6 weeks by qPCR. ∆∆CT-values of mCherry DNA normalized with genomic mouse DNA. No increase of signal was detected in injected animals compared to baseline values of untreated mice. Statistical significance was evaluated with Student’s *t* test, *p* = 0.617, *n* values are indicated in bar graphs.

To further test if the shed AAV-DNA from our biological samples is infectious, we utilized an infection assay before qPCR detection. First, we utilized GFP and mCherry plasmids as substitutes for helper plasmids to estimate double-transfection efficiency of HEK293T cells for the infection assay and observed a transfection rate of 43.95 ± 7.92% and co-transfection rates of 96.60 ± 1.23% (Fig. S3A). Then, we examined if mCherry fluorescence could serve as a potential read-out for the infection assay because the only previously published functional AAV shedding analysis in mice utilized this parameter [17]. We used similar amounts of functional AAV2/8 particles as in our biological samples but did not observe mCherry fluorescence (Fig. S3B, left). However, helper plasmids strongly increased mCherry fluorescence when utilizing high amounts of AAV2/8 validating the capability of AAV replication with the chosen parameters (Fig. S3B, right). DNA isolated from the infection assay did not contain inhibitors affecting the PCR reaction (Fig. S3C), determined by addition of functional AAV DNA to pre-injection samples isolated after the infection assay. No significant change in CT value was observed, indicating no qPCR inhibition by the infection assay sample background (rank sum test: *p* = 1). Additionally, we controlled for potential AAV degradation during the infection assay procedure (Fig. S3D) and observed a DNA decrease down to approx. 70.04 ± 1.14% (rank sum test, *p* = 0.024) This AAV degradation must be considered when interpreting post-replication values of the infection assay as evidence for replication. The analysis of the assay sensitivity of the cellular fraction demonstrated a linear range (*R*^2^ = 0.8925) between 2 × 10^5^ and 4 × 10^3^ input copies per well with a 100% detection rate (Fig. S3E). However, the samples below this range produced signals indistinguishable from background noise, indicating a limit of detection between 4 × 10^3^–10^3^ input copies/well. The assay sensitivity for the DNA isolated from the growth medium (Fig. S3F) revealed a linear range (*R*^2^ = 0.8983) from 2 × 10^5^ to 10^3^ copies/well and a reliable 100% detection rate down to 4 × 10^3^ input copies (*n* = 3), indicating a detection limit at around 10^3^ input copies/well. Conclusively, the established assay is sufficient and sensitive enough to detect AAV replication within the signal range of our collected samples (Fig. 3B).

Therefore, we subjected the samples to the infection assay to investigate the presence of functional AAV particles (Fig. 3B). We identified viral sequences in 26 of the 28 tested samples and calculated their abundance to examine potential replication. However, no sample contained AAV-DNA copies exceeding the original input copy number but rather showed a substantial decrease (feces: 9.66 ± 1.87%; urine: 14.54 ± 3.82%; saliva 6.76 ± 1.78%), thereby not providing clear evidence of shed infectious AAV particles. Consequently, we examined the livers of our AAV-injected mice for the presence of AAV-DNA as an indicator that functional AAV particles circulated in the bloodstream after CNS injection. However, we did not find an increased signal compared to non-injected control mice (Fig. 3C). Additionally, nested PCRs with HEK293T DNA isolated after the infection assay failed to detect the presence of full-length AAV genomes except in one saliva sample gathered 36 h after AAV injection (Fig. S2C). Although not directly comparable to the nested PCRs before the infection assay, this finding indicates the presence of shed full-length AAV genomes in the respective sample. Conclusively, the employed experiments confirmed that full-length AAV DNA is contained in a small fraction of collected samples but did not deliver any evidence of functional particles. However, we cannot exclude the possibility of small amounts of shed infectious particles below the detection limit.

## Discussion

Characterization of AAV shedding in animals has become increasingly relevant in recent years. It raises the question if adequate biosafety regulations are being enforced to maintain a safe and healthy environment. Although mice are important research model organisms for recombinant AAV research, this study is the first to report AAV shedding data from mice after CNS injections. Our qPCR experiments determined a shedding duration of AAV2/8 DNA after CNS injection for up to 96 h.

We assumed reduced vector shedding after CNS compared to systemic application due to transport of AAV across the blood–brain, and blood–CSF barriers. A previous study investigating systemic administration of AAV2/8 in mice detected vectors for up to 14 days in feces and up to 37 days in urine [18]. Similar findings were observed in shedding studies in macaques, where AAV2/8 DNA clearance was reported 10 days after intravenous injection [19] and only 7 days after CNS application [10]. In contrast, studies in macaques and sheep reported a longer shedding period after CNS application compared to systemic injection [16, 20, 21]. Based on the current publications, it is not clear how CNS administration of AAV2/8 influences the shedding duration. Thus, emphasizing the necessity to examine vector shedding under different experimental conditions which consider the species, application route, viral load, and serotype for adequate evaluation. To properly estimate the potential risk of AAV shedding, testing the functionality of shed particles is essential. The detection of vector DNA in body fluids does not necessarily equate to functional particles [6] as qPCR results do not provide information about form or functionality of the AAV [22]. Our data revealed a high fragmentation of AAV genomes, leading to an overestimation of shed particles with qPCR.

The results of our nested PCRs confirmed AAV DNA fragmentation, but also revealed the presence of full-length genomes in a small subset of samples, either representing uncoated AAV DNA or potentially DNA contained in intact virions or exosomes. However, the functional analysis did not produce evidence for infectious particles in any sample. Furthermore, a published biodistribution experiment with AAV2/8 demonstrated its accumulation and strong liver transduction capabilities after systemic injection [16, 23–26]. In contrast, we did not detect accumulation of AAV genomes in the liver of injected mice, indicating undetectable functional AAVs circulating in the bloodstream following CNS application. Unfortunately, published functional AAV shedding analyses in laboratory animals are rare and completely lacking in mice. One study in sheep detected AAV DNA in stool samples for up to 72 h after intravenous AAV2/8 administration, whereas functional particles were only confirmed for 48 h [16]. Functional shedding data in rabbits demonstrated AAV2 DNA in semen for 13 weeks, while infectious particles vanished after 4 days [27]. In macaques, AAV DNA was found in body fluids for up to 6 days after intramuscular administration, without identifying the presence of infectious particles [28]. Although not reported in mice, these studies emphasize that functional AAV particles can be shed under certain conditions in different species, but virions and DNA debris do not necessarily follow the same shedding dynamics.

One of the major concerns regarding AAV shedding is the therapeutic application in humans. Shedding in humans shares similarities with animal shedding in different regards and animal data can provide valuable translational insight. As in animals, shedding of viral DNA into urine, saliva, semen, and feces has been reported in humans. It can take place in a dose- and application-dependent manner and decreases over time [4, 6]. In contrast to animals, shedding of functional AAVs has not been reported in humans to our knowledge [29]. Which is surprising, since clinical trials pose a much bigger threat to the environment compared to the contained use of AAVs in laboratories. Even negative data are highly valuable in this context and should be reported.

Taken together, AAV shedding dynamics in animals seem highly variable. In our specific experimental setup, we conclude that the environmental risk after AAV CNS injection in mice is substantially low. However, we know that shedding of minuscule amounts of functional AAV particles into the environment cannot be ruled out completely. However, there have been reports that rAAVs are less infectious and stable than wtAAVs, thereby further reducing a potential environmental risk caused by AAV shedding [30, 31]. Although clear legislative regulations concerning AAV shedding is lacking, a few regulation and guidance documents exist addressing this topic directly or indirectly. Like the European Union, the NIH guidelines (App. G-II) do not mention AAV shedding specifically but require the destruction of all recombinant organisms before release [32]. Therefore, the treatment of AAV-shedding waste would depend on the presence of functional AAV particles. A guidance document of the University of California states that bedding of AAV-transduced animals must be disposed as biohazard waste for the first 72 h following vector administration, specifically including CNS injections [33]. The time period fits to our results regarding the detection of AAV DNA fragments and is likely a result of the application of the cautionary principle. In contrast, a government-associated Dutch advisory board published a statement concluding that the risks on the environment by AAV shedding are negligible, aligning with our negative results for functional AAV particles [34].

The findings in the present study should be considered when designing adequate regulatory measures by balancing theoretical safety aspects with pragmatic considerations. It is essential to maintain a reasonable and flexible regulatory framework to advance our understanding and development of therapeutic AAV-based approaches for human benefit.

### Supplementary information

SUPPLEMENTARY INFORMATION A shedding analysis after AAV8 CNS injection revealed fragmented viral DNA without evidence of functional AAV particles in mice.

## Data Availability

The data that support the findings of this study are available from the corresponding author, Melanie D. Mark, upon request.
