# Supplementary material for: A shedding analysis after AAV8 CNS injection revealed fragmented viral DNA without evidence of functional AAV particles in mice
Source: Gene Ther. 2024 Mar 12;31(5-6):345–51. doi: 10.1038/s41434-024-00447-z (PMC11090812; doi:10.1038/s41434-024-00447-z)
Supplement: Supplementary file 1 — SUPPLEMENTARY INFORMATION A shedding analysis after AAV8 CNS injection revealed fragmented viral DNA without evidence of functional AAV particles in mice. [file 41434_2024_447_MOESM1_ESM.docx]

**SUPPLEMENTARY INFORMATION**

**A shedding analysis after AAV8 CNS injection revealed fragmented viral DNA without evidence of functional AAV particles in mice.**

Felix Krause^1^, Katja Schmidtke^1^, Mailton Franca de Vasconcelos^1^, David Schmidt^1^, Beyza Cansiz^1^, Franziska Theisen^1^, Melanie D. Mark^1*^ and Max O. Rybarski^1^

^1^Department of Behavioral Neuroscience, ND7/31, Ruhr-University Bochum, Universitätsstr. 150, D-44780 Bochum, Germany

* Correspondence:

Melanie D. Mark

Melanie.mark@rub.de

Keywords: AAV, shedding, gene therapy, infectious assay, infection assay, qPCR, laboratory-safety, bedding, mice, CNS injection


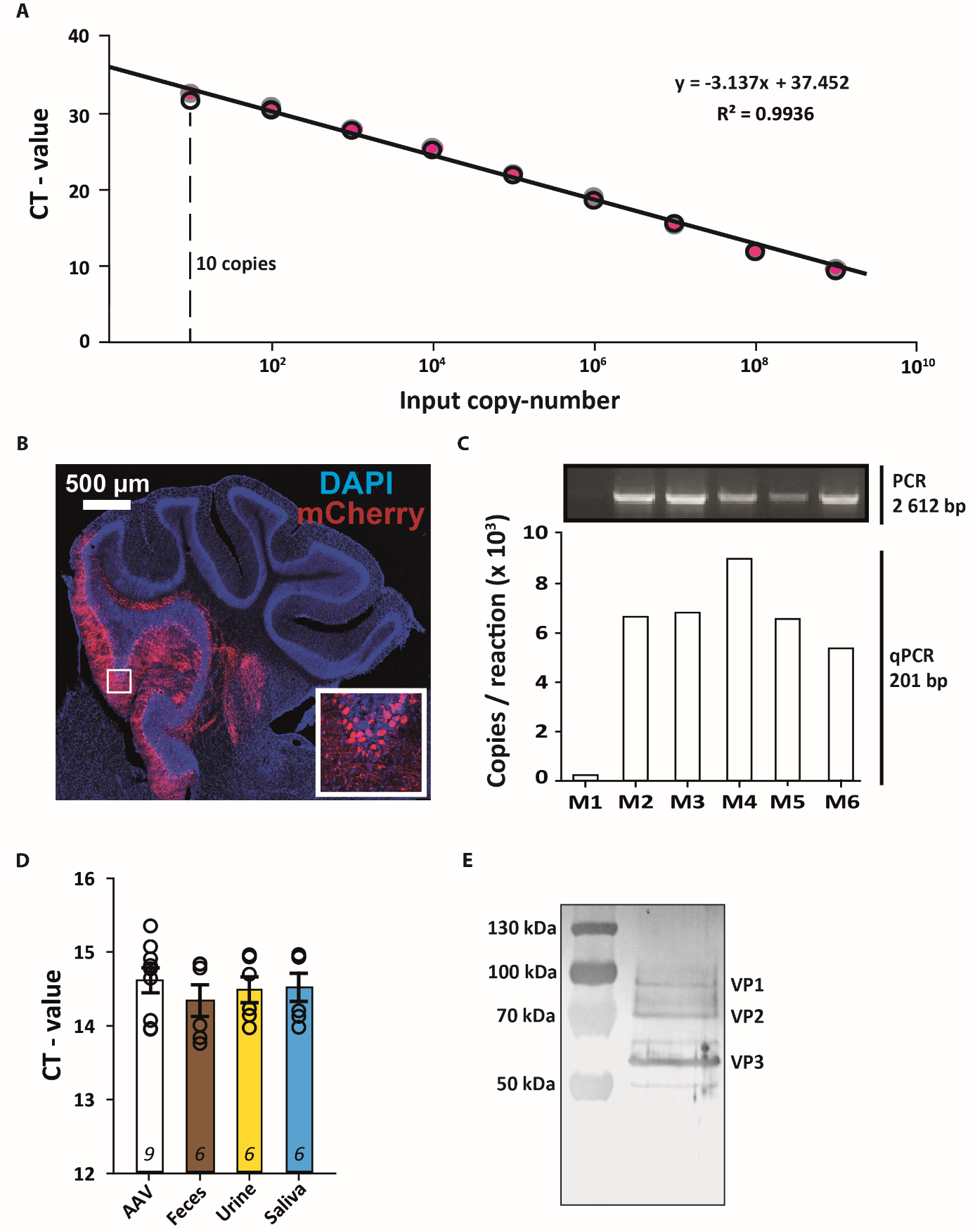


**Supplementary Figure 1:** Control experiments for the detection of viral sequences after intracranial AAV injection in mice. **A:** Dilution series of pAAV-CMV-mCherry plasmid (10^9^-10^1^ copies, triplicates=circles) reveals a quantitative range down to 10 vector copies (vc) (R^2^=0.9936. Standard curve equation to calculate vc numbers: y=3.137x+37.452. **B:** Histological image of a cerebellum injected under the same conditions as experimental animals to confirm virus expression and injection location, indicating a successful transduction of cerebellar Purkinje cells. mCherry (red), DAPI (4’,6‑diamino‑2‑phenylindole, blue) nuclear stain **C:** Quantification of AAV DNA from isolated Cerebella DNA with PCR and qPCR confirmed successful CNS delivery of the AAV. cerebella (M1-M6) were extracted 6 weeks after AAV injection. qPCR fragments (201bp) were detected in all experimental animals, although the band intensity of M1 was substantially lower (5,779±2,702 copies/reaction). AAV genome integrity was examined with PCR using a primer located in the ITR region (product size: 2,612bp). A single, correct band was detected in all experimental samples, while a low amount of vector copies in M1 was also reproduced in the PCR experiment, but the later examined shedding profile of M1 was not visibly deviating from the other samples, indicating no reduced AAV application. The low signal is likely a result of a different injection location (e.g., liquor, cortex, brain stem). The level of full-length AAV product detected by PCR differed from the amount of smaller, AAV DNA with qPCR (e.g., M4) indicating the detection of additional, fragmented AAV DNAs with qPCR. **D:** Functional AAV virus solution (1.5x10^7^) spiked with isolated pre-injection DNA of feces, urine, and saliva samples (100ng) did not display inhibition of the qPCR reaction, indicating complete removal of potentially inhibiting residues in the column-based extraction process. Statistical significance was evaluated by ANOVA on ranks, p=0.664, n values are indicated in bar graphs). **E:** Colloidal gold staining of a membrane containing separated proteins from 1µl AAV stock solution (2.1x10^9^ vc) confirms the presence of the three capsid proteins (VP1-3) and reveals slight impurities.


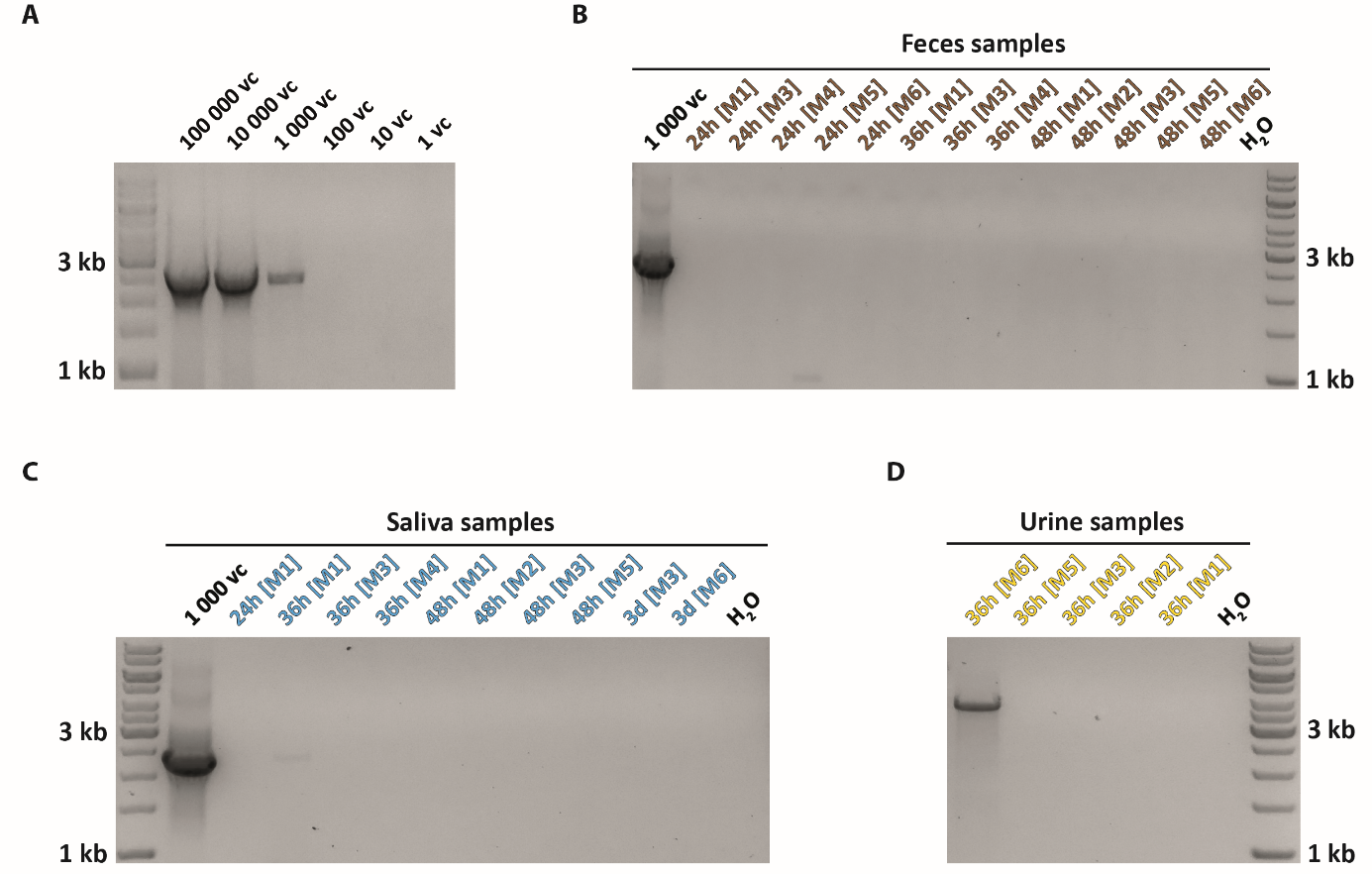


**Supplementary figure 2:** Detection of full-length AAV copies (vc) after the infection assay with nested PCR. **A:** Gel image of full-length AAV PCR products (2,448bp) from a dilution series of DNA isolated from an AAV vector stock (100,000-1 vector copies). The PCR reliably produced a band at the expected weight at least down to 1,000 AAV vector genomes as an input. **B:** Gel image of full-length AAV PCR products from HEK293T cell DNA isolated after the infection assay with collected feces samples. 1,000 AAV vc were used as a positive control. No full-length genomes could be detected in any sample. **C:** Gel image of full-length AAV PCR products from HEK293T cell DNA isolated after the infection assay with collected saliva samples. 1,000 AAV vc were used as a positive control. A weak band indicating the presence of full-lengths genomes could be detected in one sample gathered 36 hours after AAV injection. **D:** Gel image of full-length AAV PCR products from HEK293T cell DNA isolated after the infection assay with collected urine samples. 1,000 AAV vc were used as a positive control. No full‑length genomes could be detected in any sample.


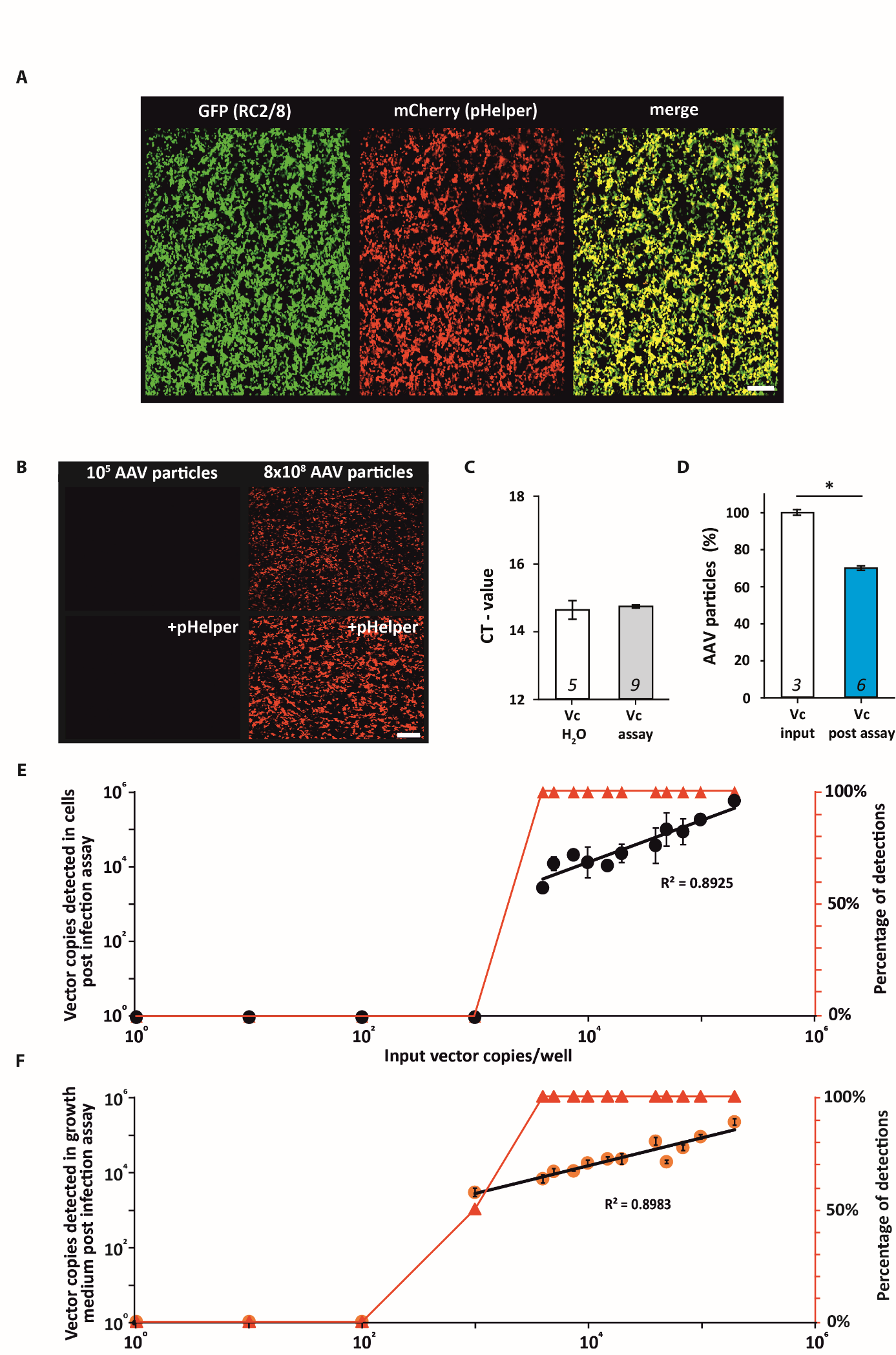


**Supplementary Figure 3:** Control experiments for the detection of functional shed AAV particles with the established infection assay. **A:** Confocal images of HEK293T cells transfected with GFP (left) and mCherry (middle). The same plasmid amounts as for RC2/8 and pHelper plasmids were used to indirectly estimate the transfection efficiency of helper plasmids based on the fluorescent proteins. Overall transfection rate was determined to be 43.95%±7.92% (mean±SEM; 2,588 cells counted). The co-transfection efficiency can be seen in the merge image on the right (96.60%±1.23%; mean±SEM). **B:** Confocal images of HEK293T cells after the infection assay. (left) Investigation if a fluorescence-based read-out of mCherry expression could be utilized to detect AAV expression or replication (+/- helper plasmids) in the particle range of the obtained bio samples (feces: 56,683–251,744; urine: 62,395–139,150; saliva 76,198–275,902. 🡪 100,000 functional particles applied in the experiment). (right) Confirmation that the established infection assay allows AAV replication determined by an increase of mCherry signal compared to AAV transduction without helper plasmids (8x10^8^ AAV particles applied). The experiment also indicates the capability of the applied virus to successfully transduce and replicate in the used conditions. Scale bar = 200 µm **C:** 1.5x10^7^ vector copies (vc) AAV DNA was quantified by qPCR in the presence of 100ng of isolated DNA from HEK293T cells compared to water as a control. Similar threshold cycle values (CT) were obtained, suggesting that HEK cell DNA or remnant chemical residues from the infection assay do not seem to have an impact on the qPCR efficiency. The sample size is displayed in the bar graph. Data is reported as mean±SEM. Statistical significance was evaluated by Mann-Whitney rank sum test, p=1. **D:** Percentage of AAV-DNA (1.5x10^9^ vc/µl) loss or degraded after the infection assay. AAV mCherry virus was diluted to 1.5x10^9^vc/µl and either processed by qPCR or incubated for 72h with HEK293T cells. Detected particles after infection assay were significantly reduced to 70.04%±1.14% compared to the input amount. The sample size is displayed in the bar graph. Data is reported as mean±SEM. Statistical significance was evaluated by Mann-Whitney rank sum test, p=0.024. **E**: The number of vector copies/well (mean±SEM) and percentage of replicates detecting vc (red triangles) from HEK293T cells (black) transfected with serial dilutions of AAV2/8-CMV-mCherry (2x10^5^ to 0 vc/well). The detected vc in the cellular fraction show a linear range from 2x10^5^ to 4x10^3^ input copies per well with a coefficient of determination of R^2^=0.8925. The sensitivity limit lies between an input of 4x10^3^ vc/well with 100% detected replicates (n=3) and 1x10^3^ vc/well (n=4) with 0% detections. **G**: The number of vc/well (mean±SEM) and percentage of replicates detecting vc (red triangles) from the growth medium (orange) of transfected HEK293T cells. The extracted DNA from growth media show linearity between 2x10^5^ and 1x10^3^ input vc/well with R^2^=0.8983. The input of 4x10^3^ vc/well resulted in 100% detection of replicates (n=3), whereas 1x10^3^ vc/well (n=4) showed 50% detects and with an input of 1x10^2^ vc/well (n=4) 0% replicates could be detected, representing a sensitivity limit of 1x10^3^ vc/well.
